# The Applied Anatomy of the Nervous System

**Published:** 1888-08

**Authors:** 


**The Applied Anatomy of the Nervous System.** Being a study of the portion of the human body from a standpoint of its general interest and practical utility in diagnosis, designed for use as a text-book and a work of reference. By AMBROSE L. RANNEY, A. M., M. D., Professor of the Anatomy and Physiology of the Nervous System in the New York Post-Graduate Medical School and Hospital, Professor of Nervous and Mental Diseases in the Medical Department of the University of Vermont, etc. Second edition. Rewritten, enlarged and profusely illustrated. 8vo., pp. 791, muslin. New York: D. Appleton & Co. Cincinnati: R. Clarke & Co. Price, $5.00.

We have already called the attention of our readers to the first edition of this excellent work. The author has succeeded in giving us all that is known of neurological anatomy, and much of the physiology of the nervous system known at the present time. So many of the phenomena of disease manifest themselves through the nervous system, that no one can be a reliable diagnostician unless he thoroughly understands the anatomy of this complicated portion of the human organism. We cannot too highly recommend this valuable work to our readers.

